# Supplementary material for: Phenopyrrolizins A and B, Two Novel Pyrrolizine Alkaloids from Marine-Derived Actinomycetes Micromonospora sp. HU138
Source: Molecules. 2023 Nov 20;28(22):7672. doi: 10.3390/molecules28227672 (PMC10675482; doi:10.3390/molecules28227672)
Supplement: Supplementary file 1 [file molecules-28-07672-s001.zip › molecules-2726485-supplementary.pdf]

Phenopyrrolizins A and B, two novel pyrrolizine alkaloids from marine-derived actinomycetes *Micromonospora* sp. HU138

Hui Zhang [1,2,3], Xiaohan Ren [1], Haiju Xu [2], Huan Qi [1], Shihua Du [3], Jun Huang [1,4], Ji Zhang [3,*] and Jidong Wang [1,*]

[1] Key Laboratory of Vector Biology and Pathogen Control of Zhejiang Province, College of Life Science, Huzhou University, Huzhou 313000, China; zhanghui_6363@163.com (H.Z.); 17729780165@163.com (X.R.); lcqhlc@163.com (H.Q.); huangj@makohs.com (J.H.)

[2] Key Laboratory of Horticultural Biotechnology of Taizhou, School of Agriculture and Bioengineering, Taizhou Vocational College of Science and Technology, Taizhou 318020, China; haijuxu@21cn.com

[3] College of Plant Protection, Northeast Agricultural University, Harbin 150030, China; dushihua12333@163.com

[4] Zhejiang Makohs Biotech Co., Ltd., Taizhou 318000, China

* Correspondence: zhangji@neau.edu.cn (J.Z.); 02752@zjhu.edu.cn (J.W.); Tel./Fax: +86-451-55190413 (J.Z.); +86-572-2321016 (J.W.)

Contents

Figure S1. The maximum-parsimony tree shown the phylogenetic relationships between strain HU138 and related species of the genus *Micromonospora* based on 16S rRNA gene sequences 3

Figure S2. The maximum-likelihood tree shown the phylogenetic relationships between strain HU138 and related species of the genus *Micromonospora* based on 16S rRNA gene sequences 3

Figure S3. The HRESI-MS spectrum of compound **1**. 4

Figure S4. The ^{1}H NMR (400 MHz, DMSO-d_6.) spectrum of compound **1**. 4

Figure S5. The ^{13}C NMR (100 MHz, DMSO-d_6.) spectrum of compound **1**. 5

Figure S6. The DEPT135 spectrum of compound **1**. 5

Figure S7. The ^{1}H-^{1}H COSY spectrum of compound **1**. 6

Figure S8. The HMQC spectrum of compound **1**. 6

Figure S9. The HMBC spectrum of compound **1**. 7

Figure S10. The HRESI-MS spectrum of compound **2**. 7

Figure S11. The ^{1}H NMR (400 MHz, DMSO-d_6.) spectrum of compound **2**. 8

Figure S12. The ^{13}C NMR (100 MHz, DMSO-d_6.) spectrum of compound **2**. 8

Figure S13. The DEPT135 spectrum of compound **2**. 9

Figure S14. The ^{1}H-^{1}H COSY spectrum of compound **2**. 9

Figure S15. The HMQC spectrum of compound **2**. 10

Figure S16. The HMBC spectrum of compound **2**. 10

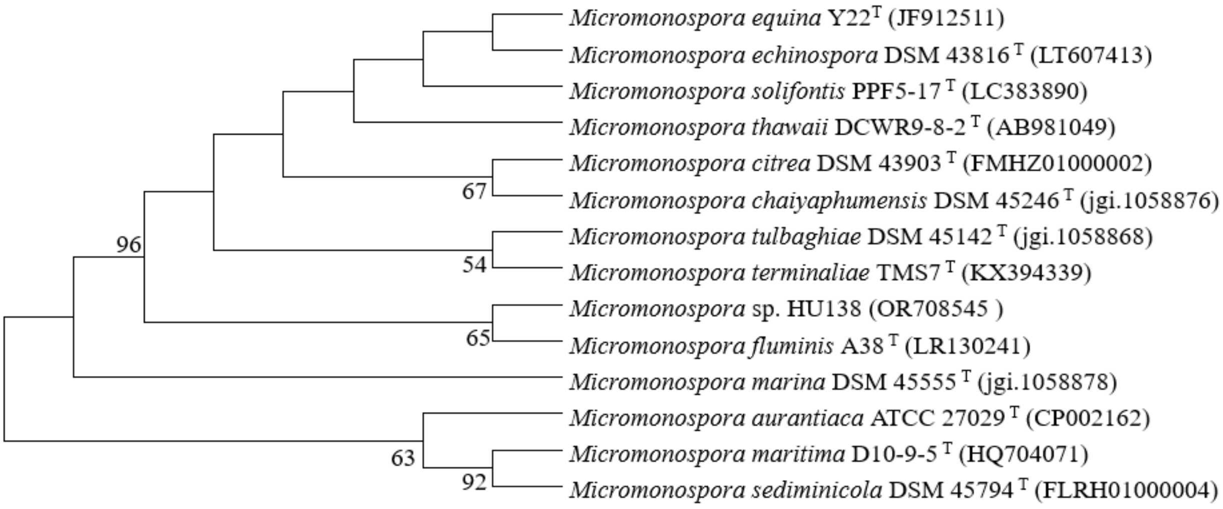

Figure S1. The maximum-parsimony tree shown the phylogenetic relationships between strain HU138 and related species of the genus *Micromonospora* based on 16S rRNA gene sequences.

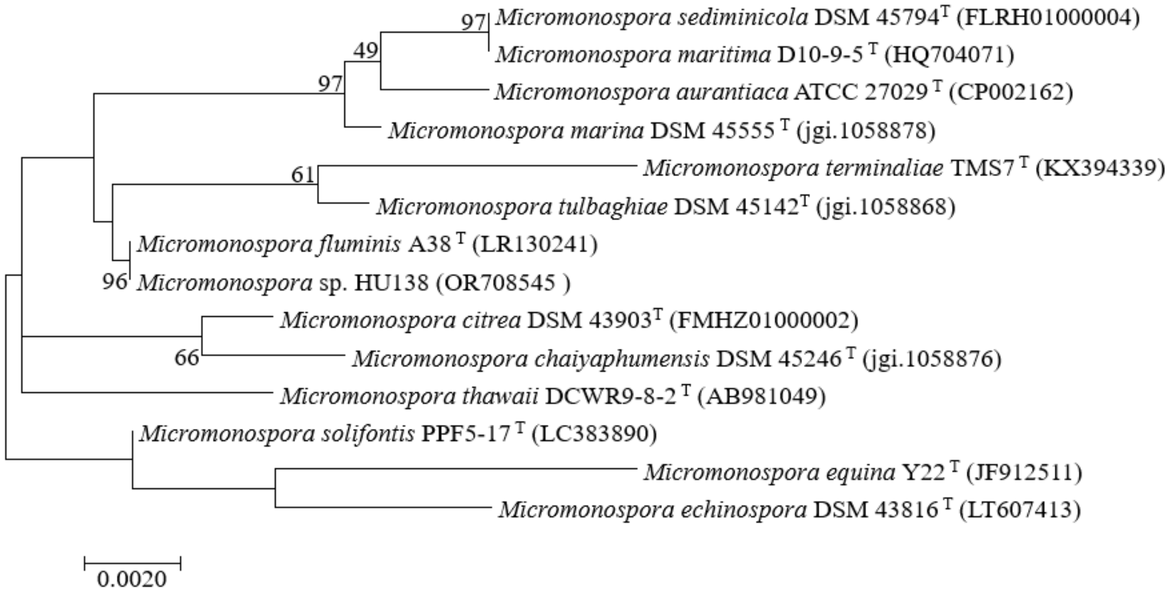

Figure S2. The maximum-likelihood tree shown the phylogenetic relationships between strain HU138 and related species of the genus *Micromonospora* based on 16S rRNA gene sequences.

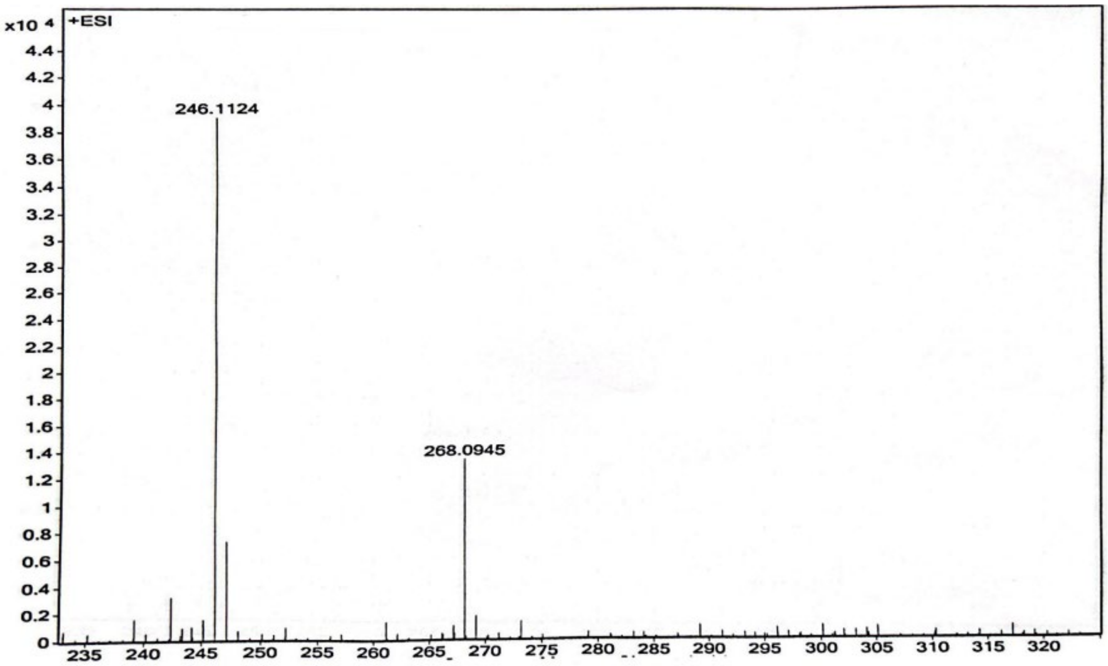

Figure S3. The HRESI-MS spectrum of compound **1**.

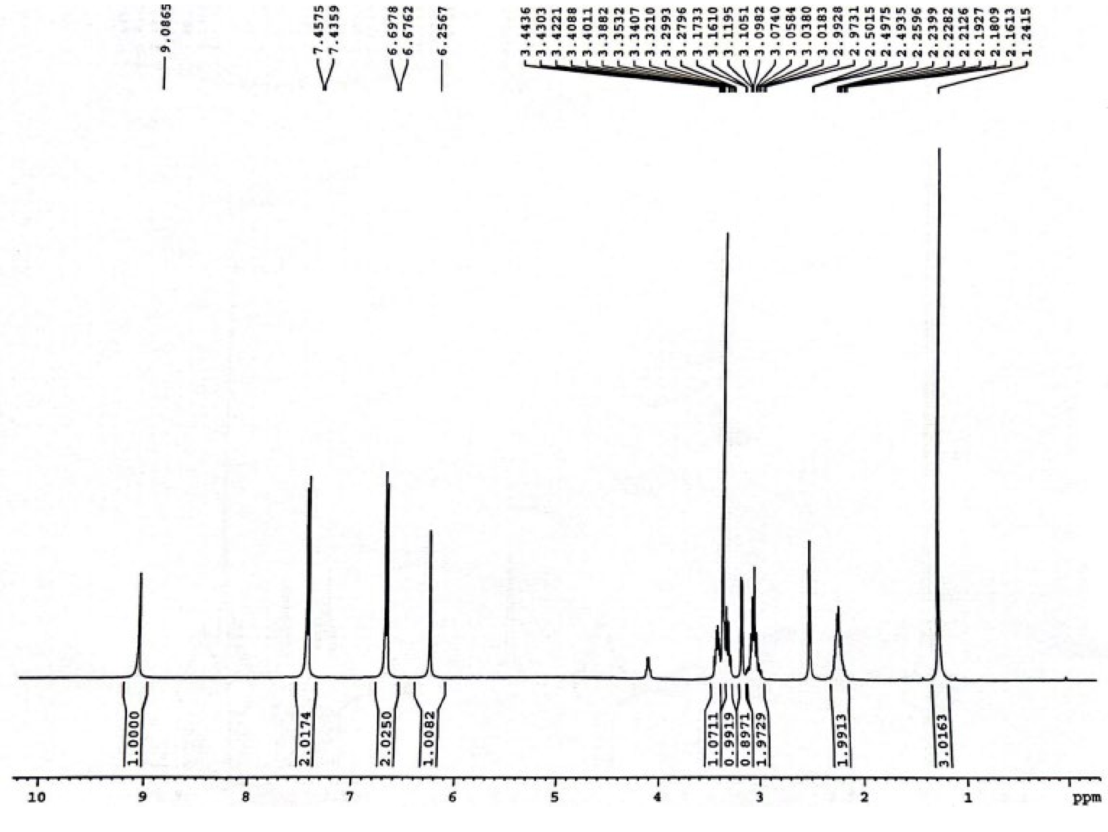

Figure S4. The ^{1}H NMR (400 MHz, DMSO-*d*6.) spectrum of compound **1**.

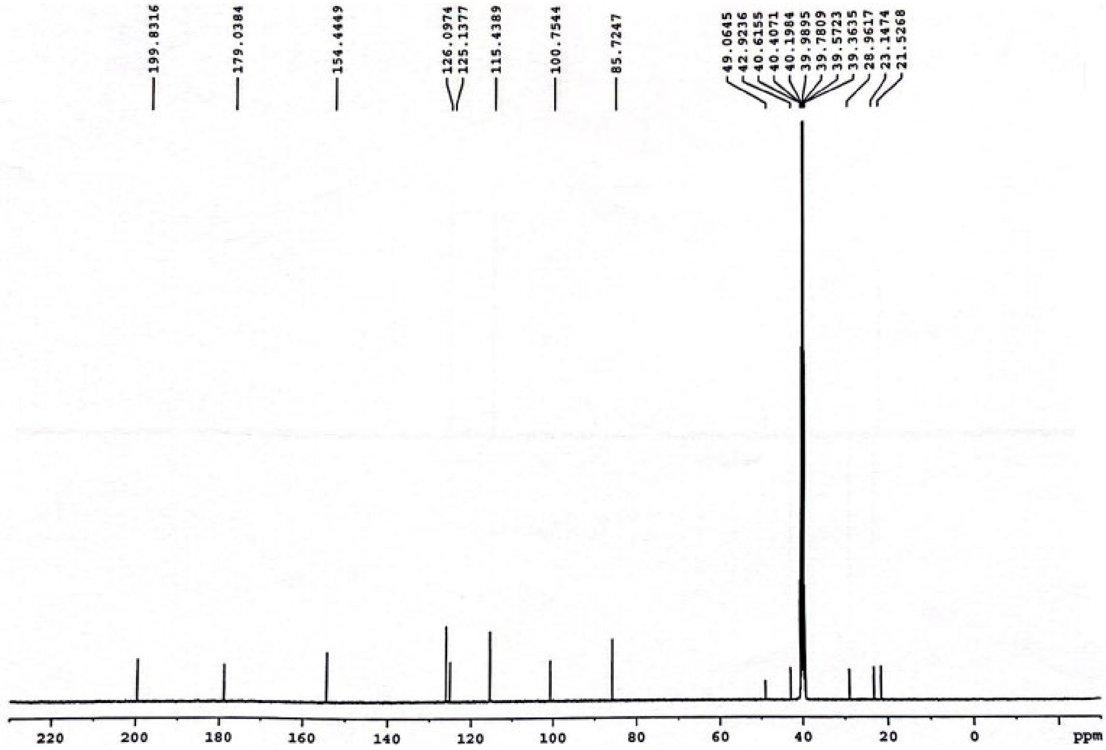

Figure S5. The ^{13}C NMR (100 MHz, DMSO-$d_{6.}$) spectrum of compound **1**.

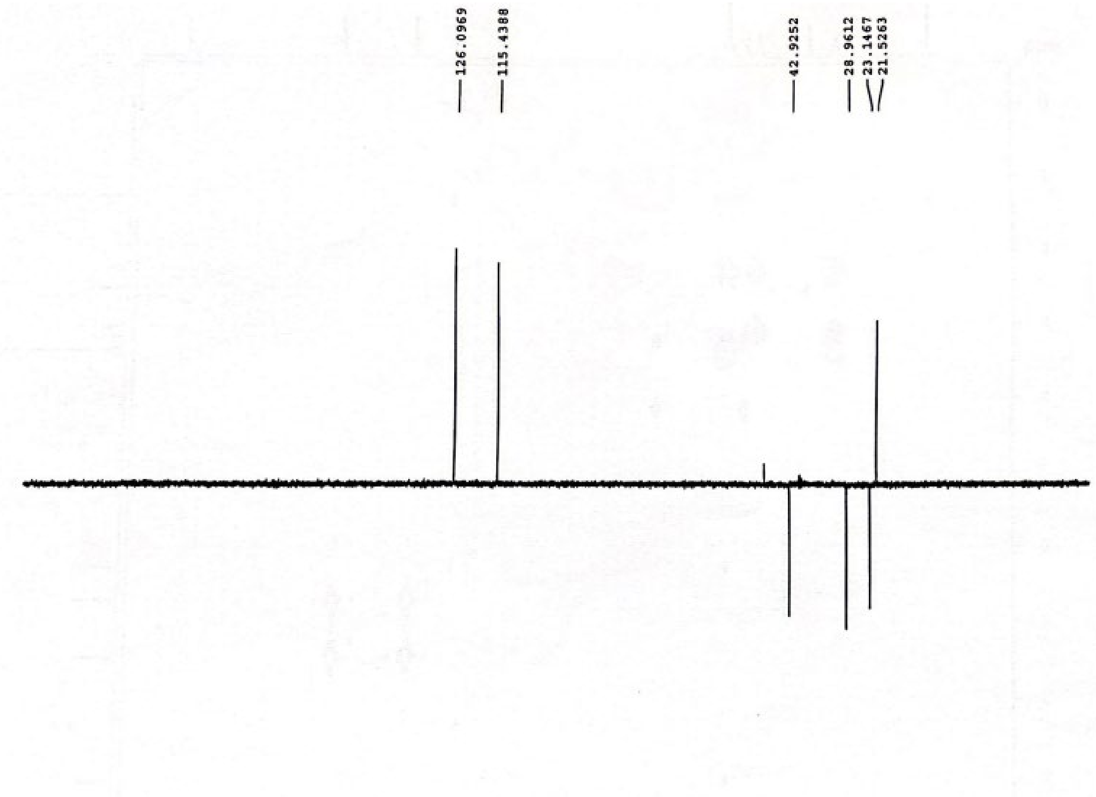

Figure S6. The DEPT135 spectrum of compound **1**.

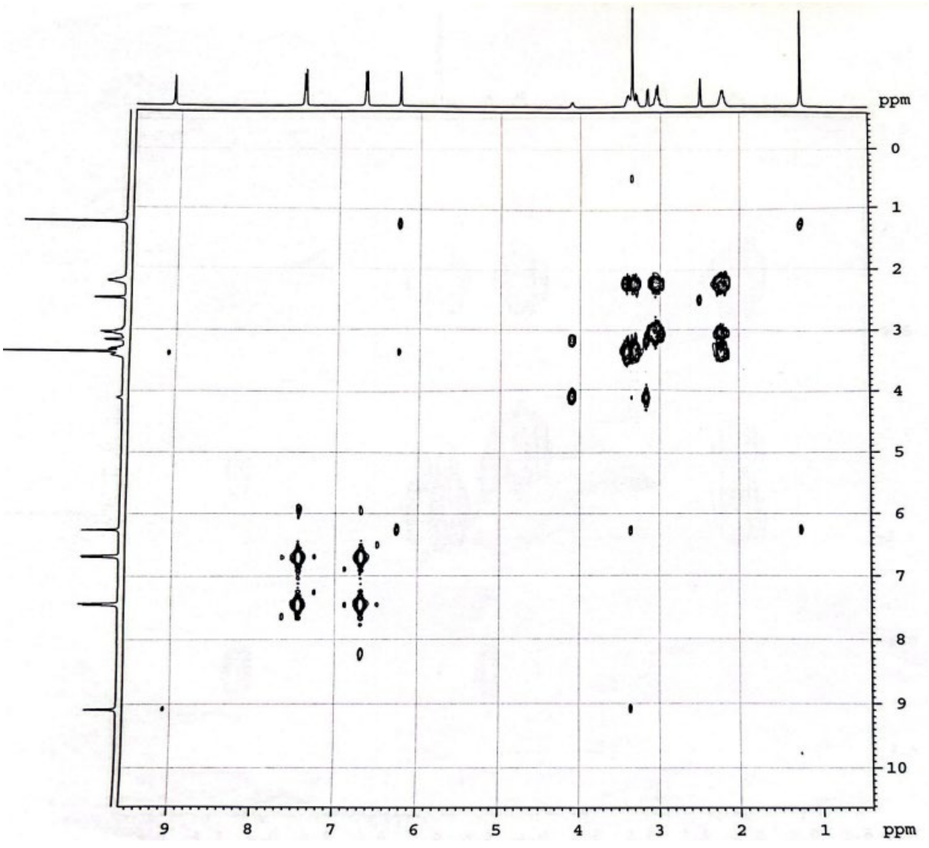

Figure S7. The ^{1}H-^{1}H COSY spectrum of compound **1**.

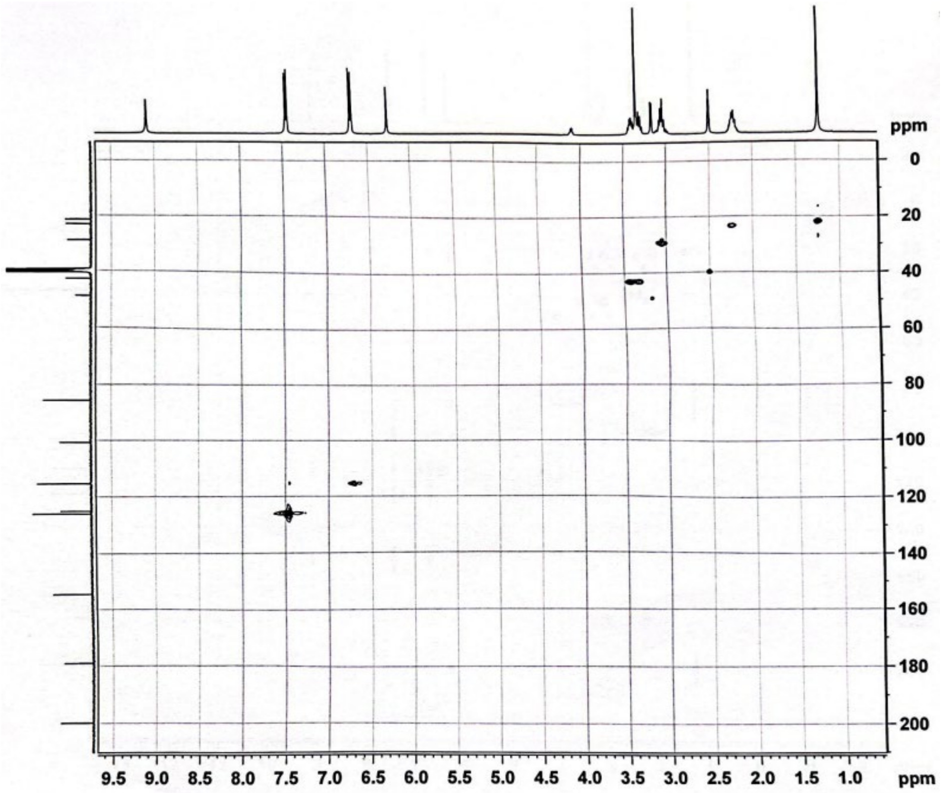

Figure S8. The HMQC spectrum of compound **1**.

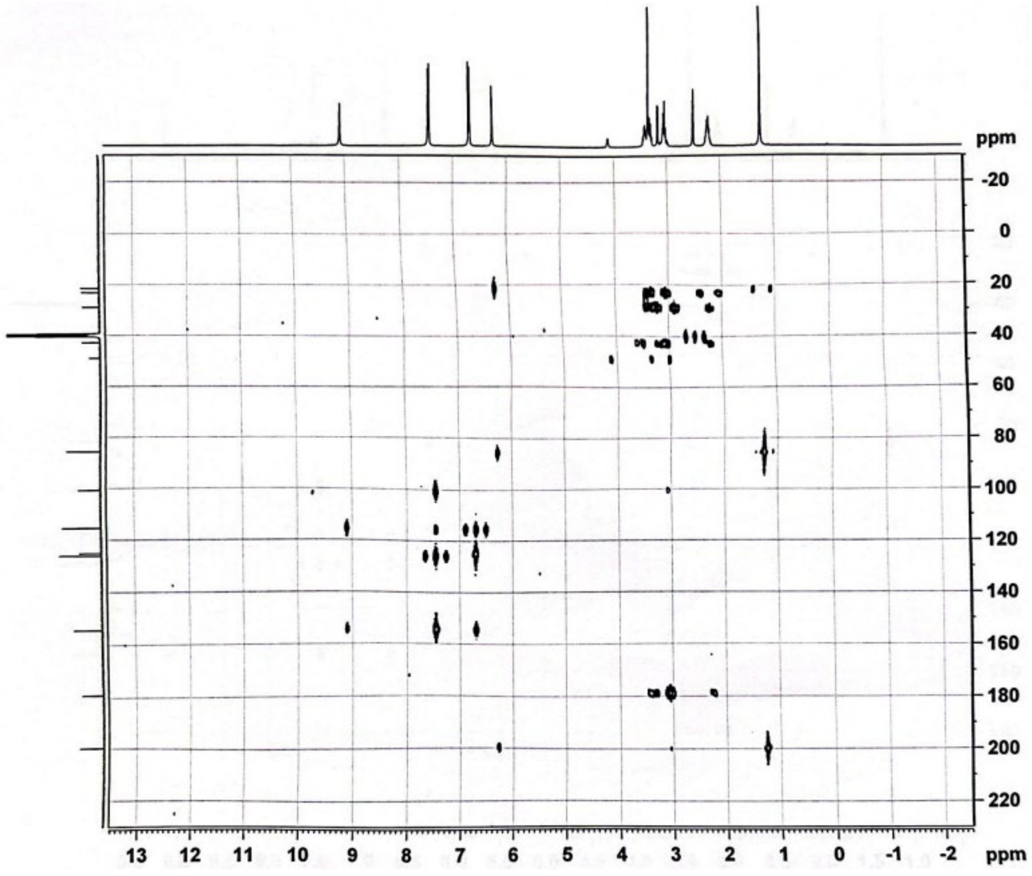

Figure S9. The HMBC spectrum of compound **1**.

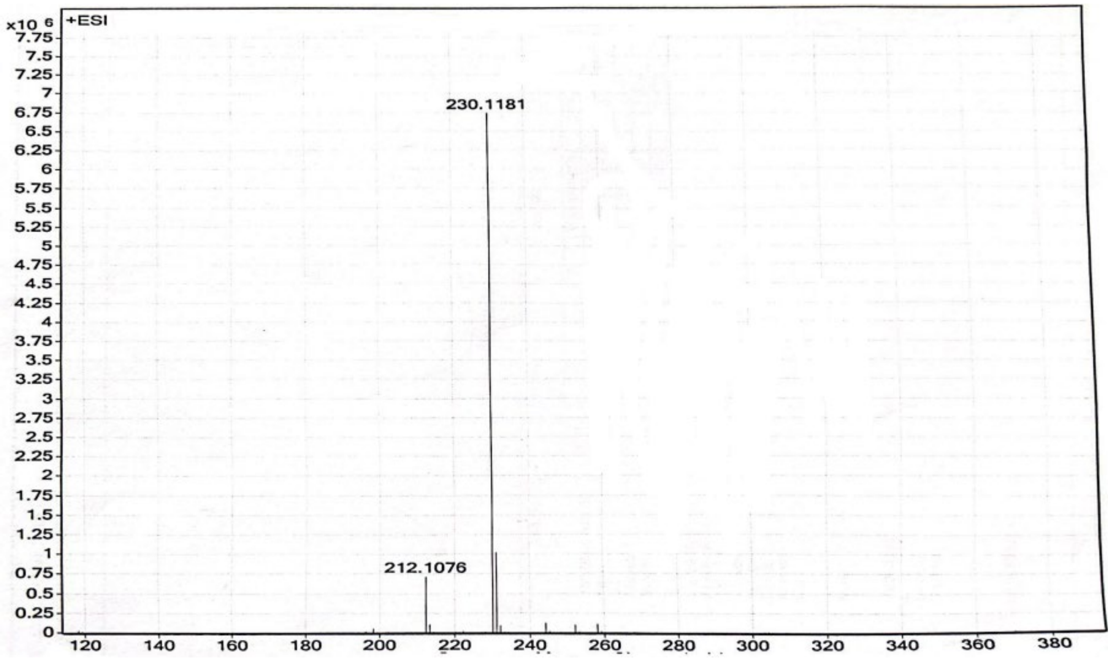

Figure S10. The HRESI-MS spectrum of compound **2**.

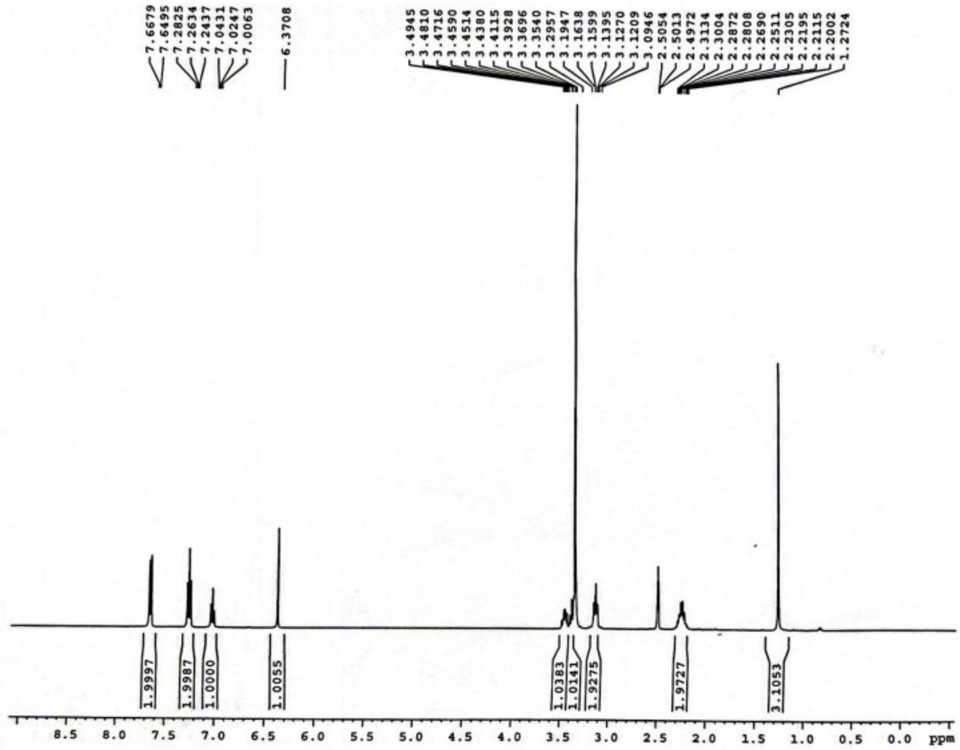

Figure S11. The ^{1}H NMR (400 MHz, DMSO-*d6.*) spectrum of compound **2**.

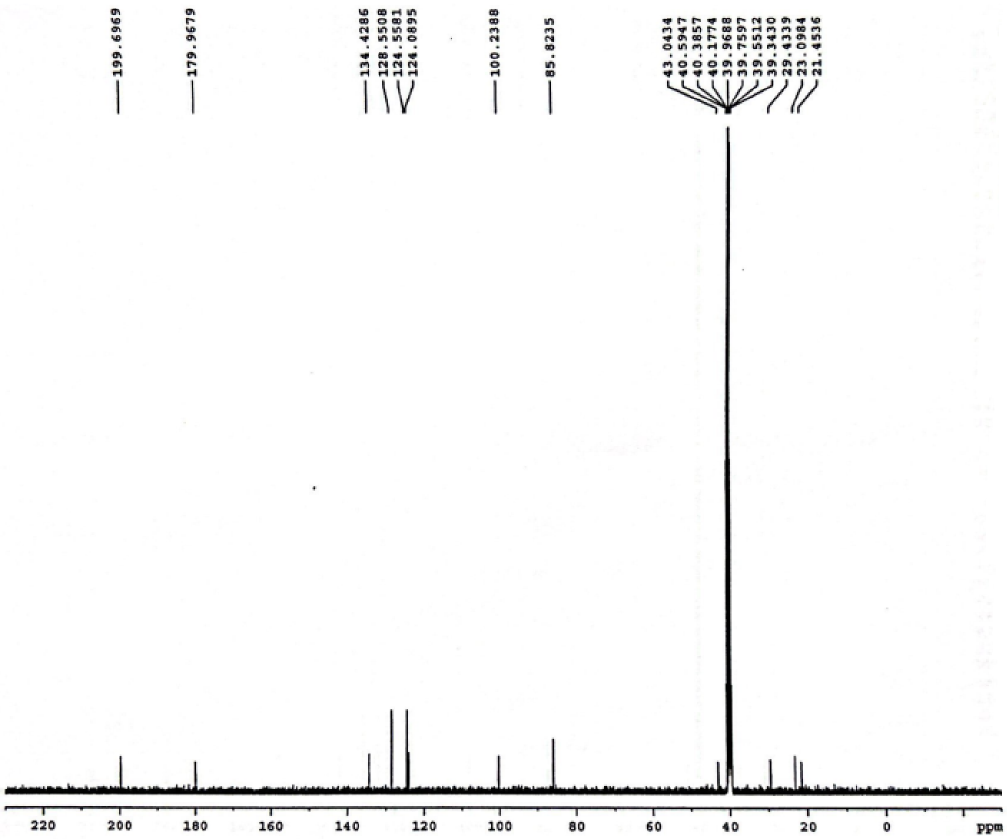

Figure S12. The ^{13}C NMR (100 MHz, DMSO-*d6.*) spectrum of compound **2**.

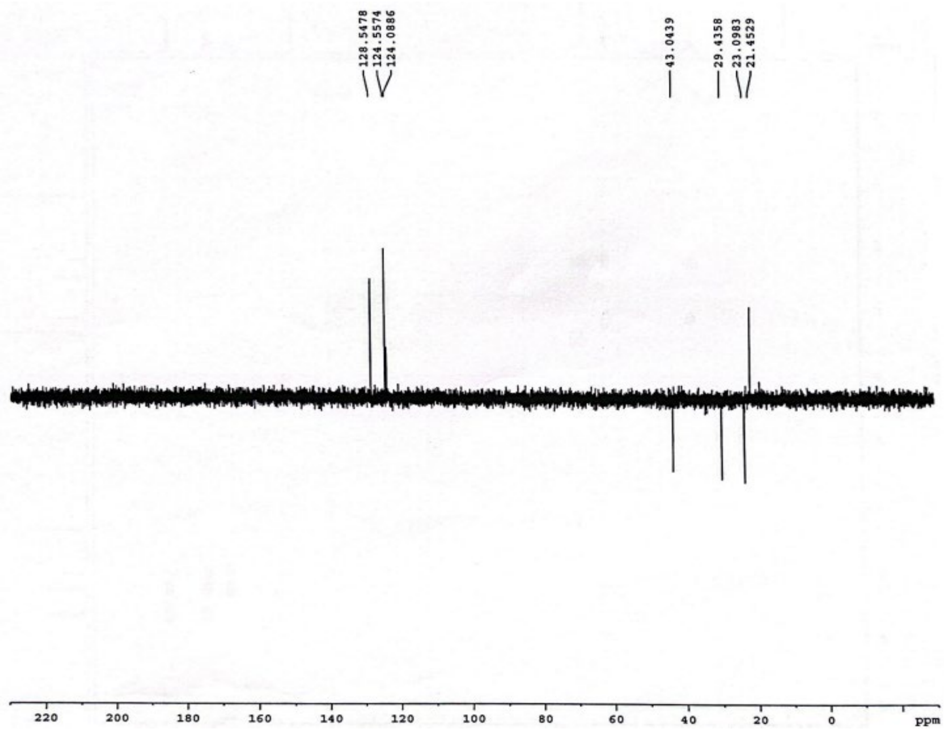

Figure S13. The DEPT135 spectrum of compound **2**.

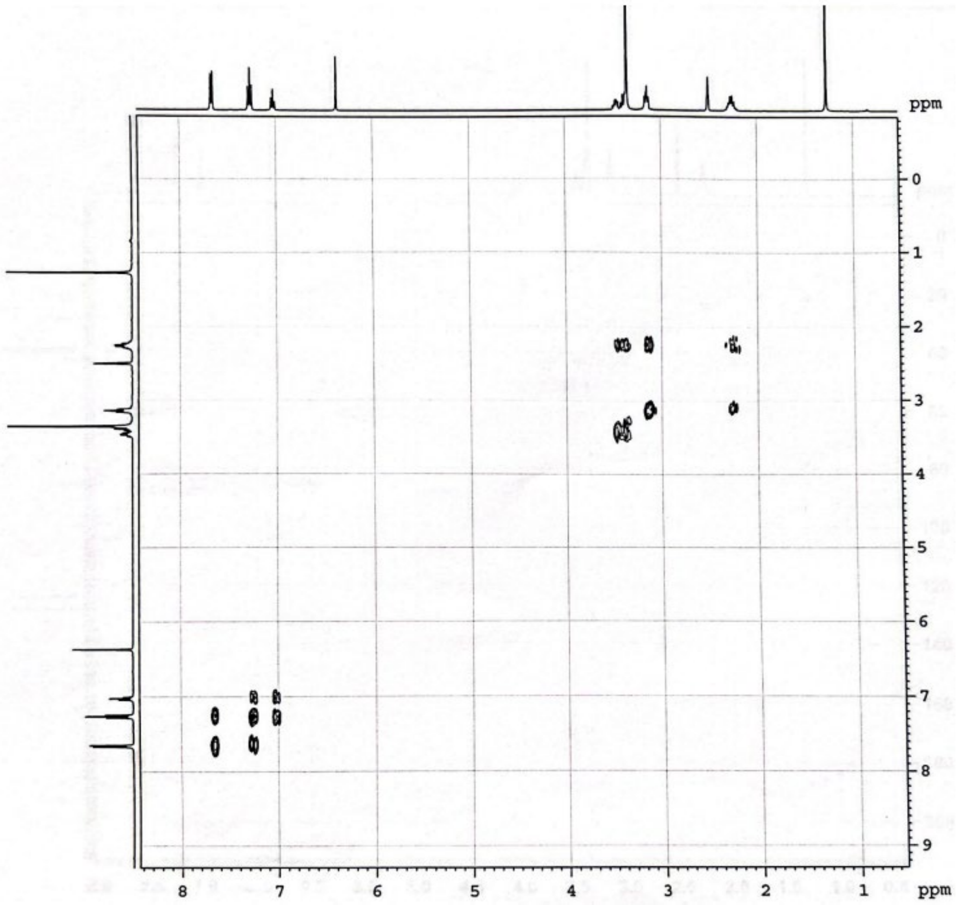

Figure S14. The ^{1}H-^{1}H COSY spectrum of compound **2**.

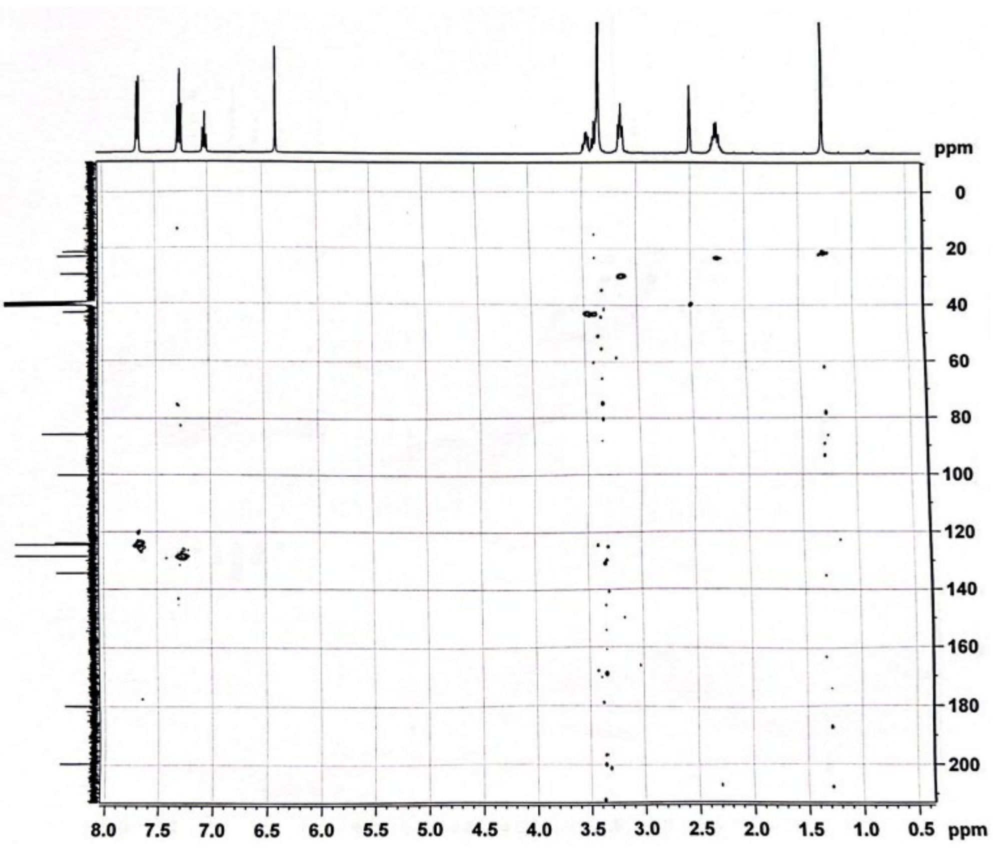

Figure S15. The HMQC spectrum of compound **2**.

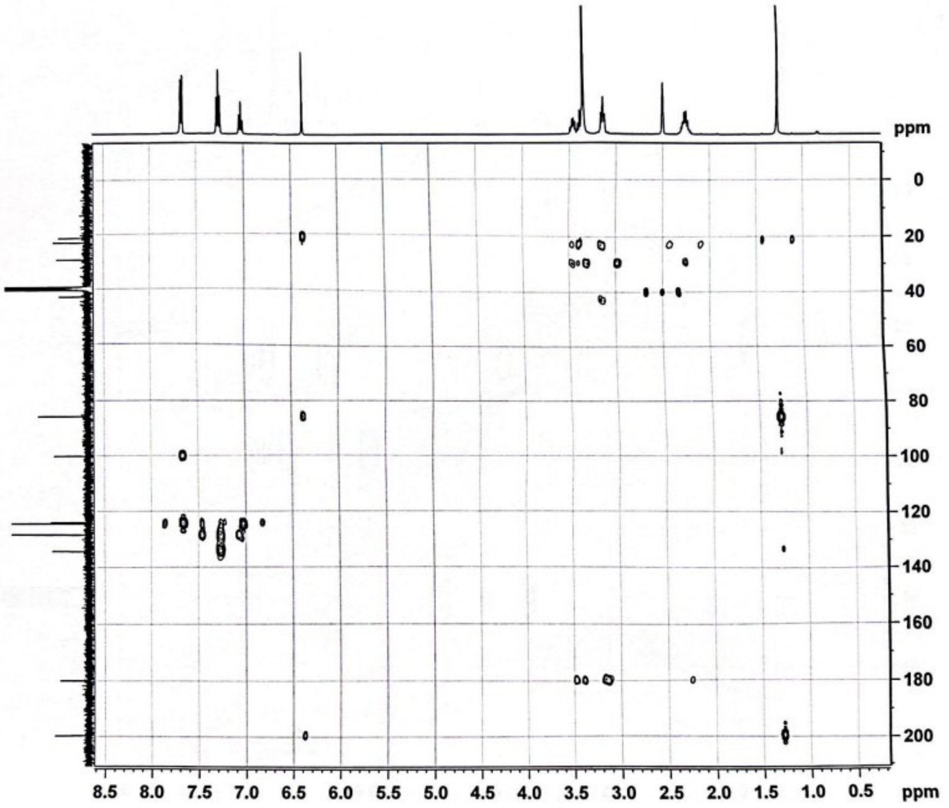

Figure S16. The HMBC spectrum of compound **2**.
